# Economic empowerment of the pilot reintegration program for female genital fistula survivors in Kenya during the COVID-19 pandemic

**DOI:** 10.3389/fgwh.2022.966390

**Published:** 2022-08-29

**Authors:** Mary Ann McCammon, Norah Amisi Otondo, Nancy Kay

**Affiliations:** ^1^US Quilts for Empowerment, Sherwood, OR, United States; ^2^Kenya Quilts for Empowerment, Luanda, Kenya

**Keywords:** female genital fistula, economic independence, COVID-19 pandemic, reintegration, psychosocial support, mentoring, Kenya

## Abstract

**Objective:**

To determine whether a pilot reintegration program for female genital fistula survivors that included a combination of financial support, psychosocial support, and mentoring would result in their long-term economic empowerment during the COVID-19 pandemic.

**Results:**

Nine fistula survivors participated in a 29-month pilot reintegration program offered by Kenya Quilts for Empowerment (KQFE), a registered Community Based Organization in Kenya. Originally, the program was intended to last for 18 months, but as a result of the pandemic, this was extended to achieve the long-term economic empowerment of women. The program was based on best practices for poverty alleviation that included multiple sources of income, psychosocial support, and mentoring, sustained over the entire 29-month period. All the women were severely impoverished at the baseline assessment, with one having some savings, and a few having productive assets, which were primarily chickens. Financial training and an initial non-refundable cash transfer provided start-up funds for small businesses; these initially flourished before floundering during the pandemic and eventually recovering. Funds were also used to buy livestock. A key component of the program was the provision of national health insurance for each woman and her family, which helped them stay healthy without having to sell any income-generating livestock. Other key components were the psychosocial support and mentoring provided within their support group. After 29 months, every woman had achieved long-term economic empowerment and “graduated” to become a KQFE ambassador, tasked with identifying fistula survivors within their communities, and referring them for surgery and participation in a reintegration support group.

## Introduction

Female genital fistula is a traumatic injury affecting up to one million women globally, including 3,000 women a year in Kenya. It is primarily caused by prolonged and obstructed labor that results in an abnormal connection between the vagina and the bladder and/or rectum resulting in continuous incontinence. Fistula survivors are impoverished and stigmatized women, often cast out of their homes, isolated, ashamed, and usually unaware that a surgical repair is possible without charge. Even after being surgically repaired, many women encounter daunting challenges when returning to their families and communities. They may be abandoned, financially exploited, and abused, while also experiencing pain, weakness, and anxiety about fistula recurrence (1–5).

A significant contribution to describing women's post-surgical reintegration is the Post-Fistula Repair Reintegration Instrument, which measures women's reintegration with their families and community during the first year after surgery (6). Exploratory factor analysis suggests four subscales and a single, higher-order latent variable of reintegration that correlates significantly with lower rates of depression and stigma, higher self-esteem, social support, and quality of life, thereby validating the concept of reintegration.

Currently, most reintegration services have been developed independently and are offered by surgical centers conducting repairs. They are primarily short-term programs offered in the immediate post-surgery period and typically include psychosocial and health counseling, physical therapy, and life-skills training. Initial benefits include improvements in physical and psychosocial health (7). While these programs are efficient and provide critical services to those women who live far from the centers and who may never return for subsequent care, their breadth and scope are insufficient to produce long-term benefits (8).

Some reintegration programs also provide measures for economic empowerment that target both short- and long-term outcomes, such as business and vocational training, income-generating activities, and micro-finance support. For example, the reintegration support groups offered by the Fistula Treatment Network in Kenya have resulted in improved income for most participants (9). However, no program has reported long-term economic empowerment leading to economic independence for women (8).

A major challenge is a mismatch between the short-term nature of current economic empowerment programming and women's needs. The literature on poverty alleviation is clear that long-term economic empowerment for people living in extreme poverty requires a holistic, multi-faceted, and long-term commitment (10). This is precisely the approach needed for fistula survivors, most of whom are heads of households living in extreme poverty.

The BOMA Graduation Model has proven that a holistic approach can successfully empower people living in ultra-poverty over a sustained period (11). This comprehensive approach includes living stipends, access to health care, business training, livelihood development, productive asset transfers, and regular mentoring. Central to this is a productive asset transfer, such as cash or livestock, coupled with extensive support that ensures benefits from this asset in the future. Living stipends and health insurance help preserve and grow productive assets rather than spending them on food or in emergencies.

In 2017, the BOMA Project and the Government of Kenya launched and evaluated a graduation pilot program to evaluate its effectiveness in lifting ultra-poor households out of extreme poverty. The key components included: cash transfers, business training, monthly stipends, free health insurance for 18 months, membership of a savings group, intensive mentoring, and social integration. In just 2 years, 1,526 women graduated out of poverty through the program (12).

Most poverty alleviation programs similar to the BOMA Project are community-based, bringing small groups together to form a business. This centralizes the training, mentoring, and evaluation of projects; however, this is not a feasible model for fistula survivors who do not come from the same community. In this case, group identity is based on the fact that they have a fistula, not that they are members of a particular village.

Fortunately, the BOMA Project was able to offer and evaluate the success of including a one-person-per-business model in addition to their group-based model. Group businesses resulted in more income diversity and faster growth. Individual businesses were more difficult to set up and keep afloat but were preferred since they required less collective decision-making. Regardless of the structure, participants reported that money was not their ultimate goal; providing food and health for their families was most important (12).

Consistent with guidelines that identify the need for longer-term data on outcomes (13), this report describes how the pilot reintegration program of Kenya Quilts for Empowerment (KQFE) adopted a holistic approach, which improved women's physical and psychosocial health and produced long-term economic empowerment. The goal was to offer the program until women became economically independent, which was intended to take 18 months. Due to the pandemic, this actually took almost 3 years, together with the women's unceasing determination to improve the lives of their families.

## Methods

The foundation for the program was established in late 2018 when KQFE's Program Director Norah Otondo, herself a fistula survivor, initiated the quilting program. By early 2019, Norah was teaching and paying nine fistula survivors to make KQFE's quilted products. At this point, KQFE decided to incorporate the BOMA Project practices (9) and methods used by other fistula support groups (3, 7, 9). A field secretary was hired to facilitate this process.

It was recognized that it would take all three components (the quilting program, the BOMA practices, and the support group), to intersect holistically and over time produce economic empowerment. These components provided the critical elements of multiple sources of income, psychosocial support, and mentoring.

### KQFE quilting program

First, Mary Ann McCammon taught KQFE's Program Director Norah how to embroider. Norah then taught other women how to make and use simple embroidery stitches to create products, including table mat sets, tote bags, and folk-art story quilts, which could be sold in the US. Women brought their work to monthly support group meetings and were paid and given new supplies to work on at home. Individual incomes ranged from $20 to 50 USD/month depending on the quantity and quality of their work. A key financial feature of the program is the support group fund. KQFE matched women's individual income through the fund that was used to pay for the reintegration program.

Up until March 2020, when Kenya was locked down due to COVID-19, women earned money by sewing, with their income matched by the support group fund. They were then paid a monthly cash stipend by KQFE, in lieu of sewing. This sum was initially $50, being reduced gradually in the spring of 2021 and ending in August 2021. No further sewing took place until late 2021 to fulfill a commission. Even then, there was limited need to pay women to make more products as there was a stockpile of these items in the US since they could not be sold during COVID-19.

### BOMA model

KQFE incorporated the BOMA Project's evidence-based best practices (11) to promote economic independence as it was recognized that the sewing program alone could not achieve this. These practices included: (a) a monthly income for daily needs; (b) financial training and coaching; (c) non-refundable cash transfers to invest in income-generating activities; (d) household health insurance; and (e) ongoing mentoring.

Income from sewing and the payment of COVID-19 monthly stipends met the requirements of the first practice. The second and third practices began in April 2019. After receiving business, savings, and budgeting training, women received a $100 non-refundable cash transfer from the support group fund to invest. KQFE also paid for a year's national health insurance (NHIF) for each family, a practice that has continued to the present. Support group funds pay the $60/year annual fee. Ongoing financial training and mentoring by KQFE's field secretary constantly emphasizes the importance of saving for insurance and other unexpected expenses.

### Support group

The women's support group is facilitated by KQFE's field secretary who also helped the group write a constitution that was registered with the government in 2019. The group has met monthly since early 2019 apart from several months during the COVID-19 pandemic. In addition to providing psychosocial counseling and social support, the group offers members two ways to access income. Members contribute small amounts of money each month to table banking and merry-go-round banking, with distribution of these funds rotated. The field secretary provides mentoring on their investments. For example, in 2021, it was arranged for an expert to provide training on animal husbandry as women's goats were dying, primarily from cold exposure at night. The field secretary also makes home visits, especially when women are sick or have a particular problem. In addition to support group meetings, women now also meet in someone's home each month to learn about how they are managing.

The women have become enduring sources of support for each other. For instance, when a young member became pregnant and her mother-in-law was insisting on a home birth, support group members accompanied her to prenatal visits to ensure she had a scheduled cesarean delivery, which was successful for both mother and baby.

### Measures

Nine fistula survivors were part of a systematic study lasting 29 months. Quantitative baseline data were collected in April 2019, and again in December 2020, and December 2021 using a program-developed questionnaire. These were orally administered in Kiswahili by phone or in person by bi-lingual female enumerators, with the answers recorded in English. Personal information included age, education, and marital status; household membership, including children and whether they were in school; and fistula experience. Asset information included sources of income from savings and small businesses, and whether the women owned land or livestock. Food security was measured by asking how many individuals they were feeding. At the start of 2020, questions were asked about whether, during the last week, all adults had at least two meals a day and if any child went to bed without an evening meal. Worries about food, clothing, household items, school fees, and “other” were scored as: no worry, small worry, big worry, or very big worry. Open-ended questions also asked about their biggest worries and hopes.

Personal empowerment was measured by asking for a yes or no response to five questions about comfort in their community, if respondents had friends, whether people visited them or they visited others, and if they went to church. Self-esteem was measured by the Rosenberg Self-Esteem Scale. Depression was measured by asking how often they felt down, depressed or hopeless. Hopefulness was measured by asking if they were very hopeful, somewhat hopeful, or not hopeful.

Qualitative data were collected twice during 2020 to determine how women were coping with the pandemic. In May, women were asked how they were managing, especially with access to food and basic needs. They were asked how they had used the April 2019 cash transfer, how they would invest a subsequent cash transfer, and what impact KQFE had on their welfare during the pandemic. In September 2020, women were asked about their general welfare and how they were using their recent cash stipends. All interviews were conducted in Kiswahili over the phone or in person by the Program Director and the data recorded in English.

## Results

### Sociodemographic characteristics

At the time of entry to the program, all women were severely impoverished. Their ages ranged from 18 to 60, with a mean of 35. Three were high school graduates and five had finished Class 7 of primary school. The youngest woman was enrolled in college for the 3 years of the study. Four were married and lived with their husbands, four lived with family members, and one lived alone. Five had children living with them who were either of school age or older. Most were small-scale farmers and/or casual laborers unable to meet most of their daily expenses. Four provided the only source of family income, four had some support from husbands, and one from parents.

Marital status and family configurations were fluid over the 3 years. Several husbands left, a few returned, some women married, and some left their partners. Three women successfully gave birth by cesarean section. During the pandemic, relatives, including grandchildren, joined the women as they had resources that their relatives did not have.

The women had lived with their fistulas from a few months to 10 years before they were repaired. Most had been repaired for at least 6 months. For the youngest, their fistulas were as a result of giving birth as teenagers, while for older women, they were due to having previous children, or they were the result of surgery or rape. Only one woman was still experiencing urinary incontinence.

### Economic empowerment

Every woman achieved measurable long-term economic empowerment. The young woman in college stayed in school and is now preparing to graduate. The remaining findings focus on the other eight women.

The women had very few assets on entry to the program. The one woman who owned land was also the only one with any cash savings. Few had any productive assets, and these were primarily a few chickens along with two goats and two cows. Eight had an M-Pesa account, the phone-based banking system, and a National ID Card. Only two were enrolled in the National Health Insurance Fund (NHIF).

The BOMA practices began in April 2019 when, after financial training, women were given an initial $100 cash transfer to invest. All chose to invest in livestock and to start or boost small businesses, like buying and reselling vegetables. Their businesses grew until the spring of 2020 when the COVID-19 pandemic made it difficult for women to sell their products. As reported earlier, to maintain the BOMA practice of ensuring a monthly income, KQFE paid each woman a $50/month stipend. In August 2020, everyone was taught how to make soap to sell, a product that people were still buying.

Responses to the qualitative interviews in May 2020 centered on the high cost and unavailability of food. Most supplemented home-grown vegetables with expensive market purchases. Several were unable to sell expensive items like charcoal and chickens. With schools closed, a few attempted to hire tutors or relatives when they had money. Women reported that $25–100 a month was needed to cover costs, with an average of $50. Their savings were between $20–110, with most women having <$35. The majority had been spent on food. Women said they would use another cash transfer to boost their businesses and buy more livestock, including cows. All thought that a further $100 investment would provide a regular income.

In September 2020, almost all women reported using the monthly stipend to buy food for the combined total of 70 people they were now feeding. A few had bought livestock, primarily chickens and goats. Four used their stipend for expenses related to significant non-COVID-19 health problems. Most women were making and selling soap for a profit.

As COVID-19 restrictions began to lift in early 2021, women started to expand their businesses and KQFE gradually reduced their $50/month stipends until they finally ceased in August. However, KQFE continued to cover the cost of health insurance. Women used a second cash transfer of $120 in September 2021 to build houses for their livestock, a recommendation made by the livestock expert to prevent their animals from dying. Some women also used part of these funds to buy garden seeds and equipment.

In September 2021, 29 months after the initial cash transfer of $100, an inventory was made of each woman's current assets. Their investments had improved by 50% or more, primarily by owning livestock. Compared to the start of the program, all the women now had several animals totaling over 200 chickens, six goats, sheep, ducks, and eight cows. Additionally, every woman had some type of small business such as selling vegetables they had grown or bought, or other items such as charcoal, soap, chips, or collected rainwater. All but one woman had cash savings and she had just used it to pay school fees. While every woman had health insurance paid for by KQFE, only two had $60 in savings to pay for a subsequent year.

In summary, women had access to multiple sources of income. In addition to investments, everyone had access to table banking and merry-go-round funds at their support group meetings. Their individual outcomes are impressive. The youngest woman is preparing to graduate college. Four women have become salaried KQFE seamstresses in addition to owning livestock and having small businesses. The eldest has become an entrepreneur with multiple businesses and a cow. Two women with young children own cows and run small businesses from home. The remaining woman is chronically ill but still has a small business and several livestock.

### Main worries

Worries about food and school fees remained the most pressing concerns, regardless of how the question was asked. Feeding over 70 people and being responsible for school fees for children and/or grandchildren was stressful. In 2020, at the height of the pandemic, three women said that not all adults had two meals a day during the past week. One of them added that school fees prevented adults from eating. By 2021, only one woman reported adults eating <2 meals a day in the past week. Fortunately, there were no reports of any child going to bed without an evening meal and all children remained in school when they were open.

### Personal empowerment

The women remained personally empowered from the start to the end of the program. All women consistently reported being comfortable in their communities and when visiting others, having friends, and hosting friends in their homes. Their scores on the Rosenberg Self Esteem Scale remained well within the range of healthy self-esteem. Total scores at the beginning of the program averaged 26 (ranging from 21 to 33) and in 2021 averaged 30 (ranging from 25 to 34). Women's personal empowerment is consistent with the concept of reintegration (6).

However, a comparison of baseline results at entry to the program with 2021 results, showed that women were more likely to report feeling down, depressed, or hopeless nearly every day in the past 2 weeks, compared to feeling down just several days a week. Despite this, when asked how hopeful they felt about the future, every woman reported feeling very hopeful at both points in time. This is consistent with what they said were their biggest hopes and dreams—to be successful, independent, and able to care for their families.

## Discussion

These findings must be interpreted in the context of the COVID-19 pandemic, which may account for women reporting feeling sad more often in 2021 as their fears about contracting COVID-19 in the absence of testing and vaccinations, the ever-changing government restrictions, and the cumulative negative impact on their businesses. COVID-19 also increased family responsibilities, including feeding more people with ever increasing food prices, resulting in the only time when some adults did not have two meals a day. As Kenyans would never deny food to anyone, especially relatives, it is likely that many more simply had less to eat.

Life slowly began returning to normal in early 2021. However, precious time and momentum had been lost and needed to be regained. Unquestionably, KQFE helped mitigate many of the consequences of the pandemic as evidenced during the qualitative interviews in May and September 2020. One woman echoed the sentiments of many: “since COVID started, I wondered how I will manage but because of your help, I have managed.” A grateful woman added, “KQFE has saved me from darkness to light.”

Despite the pandemic, KQFE's pilot reintegration program resulted in long-term economic empowerment. Every woman substantially improved her ability to earn an income and support her family, which was their initial goal. We met four of the BOMA project's graduation criteria in September 2021: (1) No child went to bed without an evening meal in the past week; (2) The value of their businesses was 25% higher than the initial cash transfer; (3) Women had two sources of income; and (4) Women were members of a savings group. We did not meet two criteria: (1) Household members had two meals a day in the last week; and (2) Participants had a minimum of $80 in savings (12).

We have speculated about the reasons why adults did not have two meals a day and hope that this will change when food prices and household membership decline. Only two women met the criteria of having $80 in savings, the entrepreneur and one of our salaried KQFE seamstresses who also has a tailoring business. For the remainder, savings were a buffer against having to sell livestock to meet their daily needs and to pay school fees. Being able to save and pay the annual $60 health insurance fee remains their biggest financial hurdle. However, savings should be understood within the context of traditional Kenyan culture that values sharing resources. Examples of how women in the program met culturally expected behavior included using savings to pay a niece's school fees and an uncle's funeral expenses.

This pilot program illustrates why a holistic and sustained approach is necessary to achieve long-term economic empowerment. In this case, success was the result of consistently providing multiple sources of income, psychosocial support, and ongoing mentoring. However, although KQFE provided these components, it was the women's hard work, resilience, and enduring hopefulness that ultimately resulted in their success.

This study was limited by the small sample size of fistula survivors from one geographic area in Western Kenya. A potential weakness was the lack of a direct data entry system as all data were orally collected in Kiswahili, translated and recorded into English by several data collectors. Another weakness was the lack of a proven model to support women's small businesses. It is not known how effective the program would have been if the pandemic had not occurred, what other obstacles may have arisen, or whether it could have been accomplished in the initially projected 18-month period.

KQFE will use this pilot program as the basis for an expanded 18-month fistula reintegration program aimed at providing women with the skills to become economically independent. It will incorporate the Street Business School (SBS) 6-month business training program, proven to be effective in lifting women out of poverty (14). It will be facilitated by our trained Kenyan staff, who will then deliver 12 months of the Boma Project model as offered in the pilot. Metrics, including those used in the pilot, as well as the Reintegration Instrument (REF) and the SBS Impact Tracker will systematically evaluate the program.

## Data availability statement

The raw data supporting the conclusions of this article will be made available by the authors, without undue reservation.

## Ethics statement

Ethical review and approval was not required for the study on human participants in accordance with the local legislation and institutional requirements. Written informed consent for participation was not required for this study in accordance with the national legislation and the institutional requirements.

## Author contributions

MM and NO conceived the project. MM reviewed the literature and drafted the manuscript. All authors revised the manuscript and approved the submitted version.

## Funding

This project was funded by Kenya Quilts for Empowerment.

## Conflict of interest

Authors MC and NK were employed by US Quilts for Empowerment. Author NO was employed by Kenya Quilts for Empowerment.

## Publisher's note

All claims expressed in this article are solely those of the authors and do not necessarily represent those of their affiliated organizations, or those of the publisher, the editors and the reviewers. Any product that may be evaluated in this article, or claim that may be made by its manufacturer, is not guaranteed or endorsed by the publisher.
